# Prostatitis and benign prostatic hyperplasia: emerging infectious diseases?

**DOI:** 10.3201/eid0301.970113

**Published:** 1997

**Authors:** B Hennenfent


### Emergence of Epidemic O'nyong-nyong Fever in Southwestern Uganda, After an Absence of 35 Years

**To the Editor:** In July 1996, an uncommon disease suspected to be O'nyong-nyong fever was recognized in the Rakai district of southwestern Uganda. It was reported to have started in June 1996. The disease spread into the neighboring Mbarara and Masaka districts of Uganda and in the bordering Bukoba district of northern Tanzania.

The initial symptoms of O'nyong-nyong fever are high fever and generalized maculopapular skin rash with crippling arthritis, primarily in the big joints, in the absence of joint effusion. Other features are lymphadenitis, eye pain and reddening with no discharge, chest pain, and general malaise. The disease is self-limiting. All age groups and both sexes are equally affected. In areas where the disease is epidemic, 60% to 80% of the people are infected, and familial clustering is found in affected households. No deaths have been reported, but two miscarriages have been associated with infection.

The Ministry of Health (Uganda), in collaboration with the Uganda Virus Research Institute, began epidemiologic and clinical investigations of the epidemic in August 1996. Acute-phase serum samples were collected from patients, and adult mosquitoes were collected from within and around patients' homes. Virus isolates were made from acute-phase serum samples from several patients by intracranial inoculation and passage in baby mice. Attempted virus isolations from mosquito specimens are in progress. Serum samples and aliquots of the virus isolates were sent to the Centers for Disease Control and Prevention, Fort Collins, Colorado, USA, for reisolation and identification. A portion of the capside and NS4 genes of the virus isolates was sequenced and identified as O'nyong-nyong virus; the virus was isolated and sequenced directly from another serum sample. Two serum samples were positive for IgM antibody to O'nyong-nyong antigen.

O'nyong-nyong virus was responsible for a similar epidemic in 1959 to 1961, which started in northern Uganda and spread south and eastward into Kenya, Tanzania, and Zambia, and then northward from Tanzania into southwestern Uganda, where it subsided. The disease has reemerged in this area after 35 years of absence.

**E.B. Rwaguma,* J.J. Lutwama,* S.D.K. Sempala,* N. Kiwanuka,† J. Kamugisha,‡ S. Okware,‡ G. Bagambisa,§ R. Lanciotti,¶ J. T. Roehrig,¶ and D.J. Gubler¶**
*Uganda Virus Research Institute, Entebbe, Uganda; †Rakai Project Uganda Virus Research Institute; ‡Communicable Disease Control, Ministry of Health, Uganda; §Ministry of Health, Rakai, Uganda; ¶Centers for Disease Control and Prevention, Fort Collins, Colorado, USA

### Prostatitis and Benign Prostatic Hyperplasia: Emerging Infectious Diseases?

**To the Editor:** In their excellent article, Molecular Approaches to the Identification of Unculturable Infectious Agents, Gao and Moore (1) point out that molecular approaches should be unleashed on diseases such as sarcoidosis, Kawasaki disease, and type I diabetes mellitus, which are thought but not proven to be infectious. The authors, however, are overlooking the more common and most likely infectious disease of unknown etiology today—prostatitis.

According to the pathologist McNeal, the prostate gland is the most commonly diseased internal organ of the human body (2). Prostatitis is the most common prostate disease, resulting in more physician visits than either benign prostatic hyperplasia or prostate cancer, according to the National Institutes of Health (3). Despite its frequency, prostatitis as a disease and as a histologic lesion is understudied (4).

By the Meares and Stamey culture localization procedure, in which the first voided urine, a midstream urine, the expressed prostatic secretions, and a final voided urine are compared, more than 90% of cases in patients with chronic pelvic symptoms are labeled as "nonbacterial" prostatitis or prostatodynia, both of which are thought to be incurable diseases (5).

The University of Washington has documented white blood cell counts as high as 38,000 per $mm^3$, in "nonbacterial" prostatitis patients (6). According to urologist Thomas Stamey, up to 50% of all men experience symptoms of prostatitis during their lifetimes (7). The prostatitis lesion was found in 40 (44%) of 91 men at random autopsy (8). In another study of 100 consecutive autopsies on men who died suddenly in automobile accidents and from other causes, the prevalence of histologic signs of prostatitis increased with age and was highest when benign prostatic hyperplasia was also present. Prostatitis was present in 22% of men under 40 years of age and in 60% of those over 40 years of age (9).

In fact, the line between benign prostatic hyperplasia and prostatitis is blurred. Prostatitis as a histologic lesion has been found in 98% of patients with benign prostatic hypertrophy (10). Microbial tests on benign prostatic hyperplasia tissue have found significant rates of infectivity. In another study, more than 70% of transurethral resection of the prostate specimens showed clinical or laboratory signs of infection (11). Benign prostatic hyperplasia and prostatitis cannot be distinguished by symptoms, and some believe that they may be the same disease.

In these days of prostate specific antigen testing, more than 50% of men who undergo biopsies for prostate cancer have a prostatitis lesion whether they have cancer or not (Gottesman et al., unpublished data; McNeal, personal communication, 1995). Prostatitis occurs at an early age, and prostate cancer decades later, in the same part of the prostate gland, the peripheral zone.

Why aren't DNA techniques being unleashed on what is apparently the most common and most purulent unknown inflammatory disease in men—an inflammatory lesion that is associated with benign prostatic hyperplasia and prostate cancer? Surely, DNA microbial testing has important implications for all three major prostate diseases—prostatitis, benign prostatic hyperplasia, and prostate cancer.

**Brad Hennenfent**
Director, The Prostatitis Foundation
Chicago, Illinois, USA

### References

1. Gao S-J, Moore PS. Molecular approaches to the identification of unculturable infectious agents DNA vaccines for emerging infectious diseases: what if? Emerg Infect Dis 1996;2:159-67.
2. McNeal JE. The prostate gland: morphology and pathobiology. Monographs in Urology 1988;9:3.
3. National Institutes of Health. The National Kidney and Urologic Diseases Advisory Board 1990 long-range plan. Bethesda (MD): U.S. Department of Health and Human Services, Public Health Service, National Institutes of Health, 1990.
4. Hennenfent BR. The economics of urological care in the 21st century [letter]. Urology 1996;47:285-6.
5. Weidner W, Schiefer HG, Krauss H, Jantos CH, Freidrich HJ, Altmannsberger M. "Chronic prostatitis" a thorough search for etilogically involved microorganisms in 1,461 patients. Infection 1991;19:S119-25.
6. Krieger JN, Egan KJ, Ross SO, Jacobs R, Berger RE. Chronic pelvic pains represent the most prominent urogenital symptoms of "chronic prostatitis." Urology 1996;48:715-22.
7. Stamey TA. Pathogenesis and treatment of urinary tract infections. Baltimore: Williams and Wilkins, 1980.
8. McNeal J. Regional morphology and pathology of the prostate. Am J Clin Pathol 1968;49:347-57.
9. Bostrom K. Chronic inflammation of the male accessory sex glands and its affect on the morphology of the spermatozoa. Scand J Urol Nephrol 1971;5:133.
10. Kohnen PW, Drach GW. Patterns of inflammation in prostatic hyperplasia: a histologic and bacteriologic study. J Urol 1979;121:755-60.
11. Riedash G, Morhing K, Brkovic D. Concentration of ofloxacin in prostatic tissue during TURP. Drugs 1993;45 (Suppl. Preprint).

### Risk Factors for Severe Leptospirosis in the Parish of St. Andrew, Barbados

**To the Editor:** Leptospirosis, an important zoonosis in most warm-climate areas, is endemic in most Caribbean countries (1). The disease was first reported in Barbados 60 years ago (2), and since 1979 has been the subject of continual study as the result of the establishment of the Leptospira Laboratory by the governments of Barbados and the United Kingdom. The annual incidence of severe leptospirosis in Barbados over the past 17 years has been approximately 11.5 cases per 100,000 population with a death rate of 13%. However, the incidence rate varies in the parishes of Barbados. For the 12-year period from 1979 to 1991, the lowest incidence rates were in St. Peter (9.5 cases per 100,000 population) and St. Michael (9.9 cases per 100,000 population), while the highest was in St. Andrew (40 cases per 100,000 population). This greater than fourfold difference in incidence rates has been attributed to differences in rainfall (3). We performed a retrospective case-control study to determine what other factors were important.

